# Gastrointestinal Health: Clinical Research and Therapeutic Innovations

**DOI:** 10.3390/life16040548

**Published:** 2026-03-26

**Authors:** Giuseppe Guido Maria Scarlata, Ludovico Abenavoli

**Affiliations:** 1 Department of Health Sciences, University “Magna Graecia”, 88100 Catanzaro, Italy; giuseppeguidomaria.scarlata@unicz.it; 2 Center for Chronic Liver Diseases, “Renato Dulbecco” University Hospital, 88100 Catanzaro, Italy

The gastrointestinal system is one of the most complex and dynamic biological interfaces between the human body and the external environment. Beyond its primary role in digestion and nutrient absorption, the gastrointestinal system is increasingly recognized as a central regulator of systemic physiology, integrating metabolic, immune, microbial, and inflammatory signals that influence multiple organs and biological pathways. Advances in clinical and translational research have significantly increased our understanding of digestive diseases, highlighting their multifactorial nature and close relationship with systemic health. This Special Issue, “Gastrointestinal Health: Clinical Research and Therapeutic Innovations”, published in *Life*, gathers a multidisciplinary collection of studies that address the key aspects of modern gastroenterology. The contributions span a wide range of topics, including gastrointestinal oncology, inflammatory bowel diseases (IBDs), hepatobiliary disorders, microbiome research, surgical management, and emerging diagnostic and therapeutic approaches. Several studies focus on gastrointestinal malignancies and tumor biology. Real-world clinical data on total neoadjuvant therapy in rectal cancer provide insights into gastrointestinal toxicity and the predictors of treatment outcomes [1]. In addition, the COVID-2019 pandemic seriously affected cancer care worldwide, and its impact on gastric cancer management highlights the need for adaptive clinical strategies during global health crises [2]. Additionally, tumor biology and diagnostic biomarkers remain central topics in gastrointestinal oncology. An exploratory immunohistochemical study evaluated Ki-67 expression and tumor size, contributing to our understanding of proliferative activity in small bowel tumors [3]. Other studies addressed rare and complex oncological scenarios, including synchronous ileal metastasis from pancreatic ductal adenocarcinoma and diagnostic challenges related to neuroendocrine tumors in liver transplant recipients [4,5]. Together, these investigations underline the importance of integrating molecular, pathological, and clinical perspectives in gastrointestinal oncology.

The gut microbiota plays a fundamental role in maintaining host homeostasis and influencing metabolic and immune pathways. Advances in metagenomic technologies have enabled deeper characterizations of microbial communities. Specifically, a shotgun analysis of gut microflora in healthy older adult individuals produced valuable insights into microbial diversity and community structure across populations [6]. These findings contribute to establishing reference microbial profiles that may help interpret the microbiome alterations associated with gastrointestinal and systemic diseases. Acute gastrointestinal conditions remain a major cause of morbidity and hospitalization worldwide. Several studies provided updated clinical insights into the management of gastrointestinal bleeding. In this context, retrospective analyses of variceal and non-variceal upper gastrointestinal bleeding provide valuable epidemiological data, and the researchers proposed updated management algorithms to improve patient outcomes [7,8]. In addition, hepatobiliary and pancreatic diseases were addressed in studies focusing on surgical and clinical management. A study on bile duct injuries following cholecystectomy shed light onto surgical repair strategies and outcomes in specialized referral centers [9]. Similarly, an epidemiological investigation of biliary acute pancreatitis contributes to a better understanding of disease patterns and risk factors in tertiary care settings [10]. Surgical advances are further highlighted in studies comparing laparoscopic and open distal pancreatectomy, emphasizing the importance of minimally invasive approaches in pancreatic surgery [11].

IBDs represent a central area of research in gastroenterology. Several articles address different aspects of disease pathophysiology and clinical management. Psychological factors such as depression may seriously affect health-related quality of life in patients with ulcerative colitis (UC), highlighting the need for integrated multidisciplinary care [12]. Other contributions explored the specific pathological mechanisms associated with intestinal inflammation [13]. Additionally, lifestyle and nutritional factors are increasingly recognized as modulators of intestinal inflammation. In this context, a case–control study examining fruit and vegetable consumption highlighted the influences of diet on IBD risk [14]. Nutritional status assessment is also an important aspect of IBD management, as illustrated in a study evaluating phase angle as a complementary tool for assessing nutritional status in patients with UC [15].

Gastrointestinal diseases are increasingly recognized as part of broader systemic networks involving metabolic and inflammatory pathways. A growing body of evidence highlights the concept of inter-organ communication axes. In this Special Issue, the gut–kidney axis is explored through a review examining the relationship between IBDs and nephropathies, emphasizing the role of immune activation and metabolic pathways linking intestinal and renal disorders [16]. Moreover, case studies and pilot investigations evaluated metabolic-dysfunction-associated steatotic liver disease in rare genetic conditions and in patients with IBDs [17,18]. These findings underscore the complex interplay among intestinal inflammation, metabolic dysregulation, and hepatic pathology.

Another important theme in this Special Issue concerns innovative therapeutic approaches and clinical management strategies. A systematic review evaluated the efficacy of biologic therapies, such as dupilumab, yielding additional insight into emerging treatment options for eosinophilic esophagitis [19]. Novel perioperative management strategies were also investigated, including regional anesthesia techniques aimed at facilitating early extubation following liver transplantation [20]. Additional clinical innovations focused on alternative therapeutic approaches in complex medical cases. For example, the rectal administration of rifampicin and isoniazid suppositories represents a novel strategy for treating tuberculosis in patients with multiple comorbidities and gastrointestinal limitations [21]. Case reports and translational investigations further enrich this research field by addressing rare clinical conditions and emerging diagnostic approaches. These include the management of a superior mesenteric artery aneurysm associated with pancreatic cyst rupture and the use of inflammatory indices as potential biomarkers for evaluating the neoplastic potential of colon polyps [22,23]. Furthermore, experimental and translational studies also contributed to the understanding of hepatic injury and protective mechanisms. Researchers evaluated the hepatoprotective effects of *Camellia sinensis* in experimental models of cisplatin-induced liver injury, highlighting the potential therapeutic applications of natural compounds [24]. Finally, innovative clinical approaches were explored in a case study investigating functional neurology therapy in a patient with lactose intolerance [25]. Future research will increasingly rely on integrating clinical investigations, molecular biology, microbiome science, and pathophysiology to further elucidate the complex mechanisms underlying digestive diseases. Such interdisciplinary efforts will be essential for developing personalized diagnostic strategies and innovative therapeutic interventions to improve patient outcomes. In conclusion, this Special Issue, “Gastrointestinal Health: Clinical Research and Therapeutic Innovations”, provides a comprehensive overview of the current progress in the field of gastroenterology. The presented articles not only deepen our understanding of digestive diseases but also open new perspectives to developing innovative diagnostic and therapeutic strategies oriented to improve human health.

## Data Availability

No new data were created or analyzed in this study. Data sharing is not applicable to this article.

## Abbreviations

The following abbreviations are used in this manuscript:

IBD	Inflammatory Bowel Disease
UC	Ulcerative Colitis
